# Supplementary material for: High Prevalence of Staphylococcus aureus Enterotoxin Gene Cluster Superantigens in Cystic Fibrosis Clinical Isolates
Source: Genes (Basel). 2019 Dec 12;10(12):1036. doi: 10.3390/genes10121036 (PMC6947208; doi:10.3390/genes10121036)
Supplement: Supplementary file 1 [file genes-10-01036-s001.pdf]

Appendix A

Supplemental method: Genotyping assay.

Genomic DNA was isolated from overnight cultures of *S. aureus* using commercial kits following manufacturer's instructions. PCR reactions were performed with the following oligonucleotide primers, in 5′ to 3′ sequence. All PCR products were < 1 kb in size.

sea fwd GATTCACAAAGGATATTGTTGATAAATAT, *sea* rev GTCCTTGAGCACCAAATAAATC,
seb fwd GTATGATGATAATCATGTATCAGCAA, *seb* rev CGTAAGATAAACTTCAATCTTCACAT,
sec fwd GAGTCAACCAGACCCTATGCC, *sec* rev CGCCTGGTGCAGGCATC,
sed fwd GCATTACTCTTTTTTACTAGTTTGGTA, *sed* rev CCTTGCTTGTGCATCTAATTC,
see fwd CTGAATTACAAAGAAATGCTTTAAGC, *see* rev GCCTTGCCTGAAGATCTA,
seg fwd AATTCCCAACCCGATCCTAAAATAG, seg rev TCAGTGAGTATTAAGAAATACTTCCATTTTAATAC,
sel-h fwd TCACATCATATGCGAAAGCAG, *sel-h* rev TAGCACCAATCACCCTTTCC,
sel-i fwd CAAGGAGATATTGGTGTAGGTAAC, *sel-i* rev TTAGTTACTATCTACATATGATATTTCAACATC,
sel-k fwd TGGATCAATGGAAATCAACAAAA, *sel-k* rev CGGGCTACCCGAAAAATAAT,
sel-l fwd CTGTTTGATGCTTGCCATTG, *sel-l* rev GCGATGTAGGTCCAGGAAC,
sel-m fwd GATGTTGGAGTTTTGAATCTTAGGAAC, *sel-m* rev TCAACTTTCGTCCTTATAAGATATTTCTACATC,
sel-n fwd GATGTAGACAAAAATGATTTAAAGAAAAAATC,
sel-n rev TTAATCTTTATATAAAAATACATCGATATGATAATTAG,
sel-o fwd AATGAAGAAAATCCTAAAATTGAGG , *sel-o* rev TTATGTAAATAAATAAACATCAATATGATAGTC,
sel-p fwd ACCAACCGAATCACCAGAAG , *sel-p* rev GTTCAAAAGACACCGCCAAT,
sel-q fwd GATGTAGGGGTAATCAACCTTAG, *sel-q* rev CTCTCTGCTTGACCAGTTCC,
sel-r fwd TACTATGGGGAATGTTGAATCC, *sel-r* rev GGTATAAAGGGAACCAAATCC,
sel-s fwd CTAACTCTTGAATTGTAGGTTCC, *sel-s* rev CTCCACACAACTATTATCAAACG,
sel-t fwd TCGGGTGTTACTTCTGTTTGC, *sel-t* rev GGTGATTATGTAGATGCTTGGG,
sel-u fwd AAACCAGAACAATTGAATAAAGCG, *sel-u* rev TTATTTTTTGGTTAAATGAACTTCTACATTAATAG,
sel-x fwd TCTATGGGGGAACATTTGGA, *sel-x* rev CCGCCATCTTTTGTATTTATGA,
tstH fwd GAAATTTTTCATCGTAAGCCCTTTGTTG, *tstH* rev TTCATCAATATTTATAGGTGGTTTTTCA.

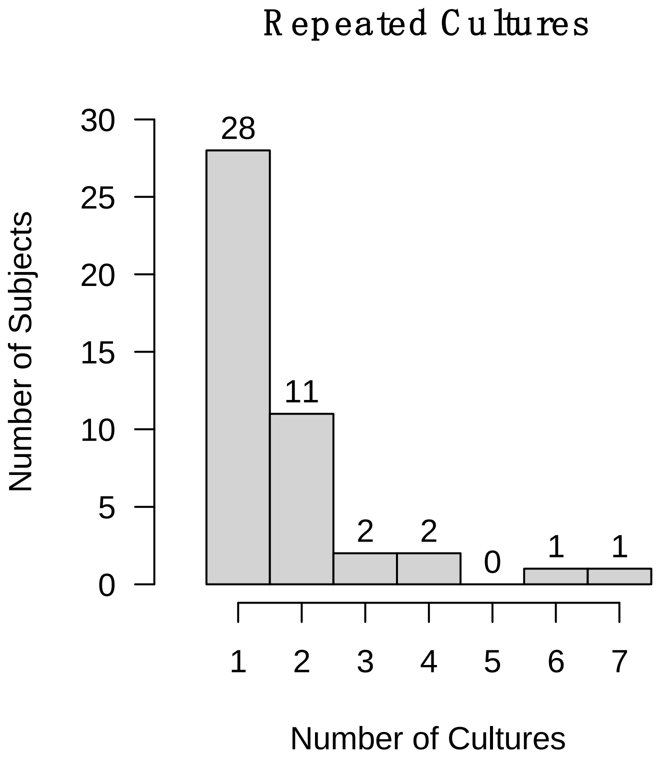

Figure S1. Frequency of repeated cultures within University of Iowa Biobank.
